# Assessment of inner ear morphology and function in response to local positive pressure for Ménière’s disease: a nonrandomized controlled trial

**DOI:** 10.1038/s41598-022-25321-z

**Published:** 2022-12-01

**Authors:** Munehisa Fukushima, Shiro Akahani, Hidehiko Okamoto, Noriaki Takeda, Hidenori Inohara

**Affiliations:** 1 Department of Otolaryngology and Head and Neck Surgery, Kansai Rosai Hospital, 3-1-69 Inabaso, Amagasaki, Hyogo 660-8511 Japan; 2 Department of Otolaryngology and Head and Neck Surgery, Graduate School of Medicine, Osaka University, Osaka, Japan; 3 Department of Physiology, School of Medicine, International University of Health and Welfare, Chiba, Japan; 4 Department of Otolaryngology, University of Tokushima School of Medicine, Tokushima, Japan

**Keywords:** Neurological disorders, Inner ear

## Abstract

Ménière’s disease (MD) is an inner ear disorder in which the main pathological feature is endolymphatic hydrops (EH). Positive pressure therapy (PPT) using a portable device is now a second-line therapy for intractable MD when initial medical treatment fails. However, it remains unknown whether PPT causes the morphological and functional changes of inner ear in patients with active MD in accordance with reduction of vertigo attacks. In this nonrandomized controlled trial of 52 patients with MD, the volume of EH significantly decreased with reduction of vertigo attacks during 8 months of PPT combined with medications while the volume of that significantly increased with medications alone. There was no difference between Control group (n = 26) and PPT group (n = 26) regarding the vertigo control, however, PPT group achieved a significant functional improvement of vertical semicircular canals. The effect of volume reduction by PPT has been firstly demonstrated and the functional changes of all semicircular canals during PPT have been firstly examined. Morphological and functional changes in the inner ear by administrating local positive pressure are quite different from those caused by medications alone.

**Clinical trial registration**: UMIN-CTR UMIN000041164 (registered on July 20, 2020).

## Introduction

Ménière’s disease (MD) is an inner ear disorder. Its symptoms are attacks of vertigo with combinations of fluctuating hearing loss, tinnitus and aural fullness in the affected ear, with a lifetime prevalence of approximately 0.5%^1^. Endolymphatic hydrops (EH) is determined in more than 98% of patients with MD, and MD symptoms and EH are thought to be closely related^2^. Some therapies for MD symptoms are aimed at reducing EH, and positivsupple pressure therapy (PPT) using a portable device is now a second-line therapy for intractable MD when initial medical treatment, such as with diuretics and steroids, fails^3^. The PPT device is designed to generate positive pressure pulses to influence inner ear fluids through the middle ear, to decrease EH volume. Relative positive pressure in the middle ear when exposed to a lowered pressure chamber was shown to relieve acute MD symptoms^4^, and the effects of local middle ear pressure application for advanced MD symptoms were reported during a 2-year follow-up^5^. A randomized controlled study conducted in 2004 using the Meniett device demonstrated decreased vestibular symptoms in the treatment group vs the control group^6^. However, previous meta-analyses have shown that the effect of PPT using the Meniett device for relieving vertigo attacks is controversial^7–9^. More importantly, however, hypothetical morphological changes in EH in vivo during PPT, i.e., decreased EH volume, have not been well investigated.

In the present study, we proposed two interventions: medications alone and medications combined with a PPT device, to confirm whether PPT causes morphological and functional changes in the inner ear in patients with active MD in accordance with a reduction in vertigo attacks. EH, an objective index of treatment effect, is currently easily visualized using 3-Tesla magnetic resonance imaging (MRI) after intravenous administration of gadolinium (Gd)^10^. Using this imaging method, we recently characterized EH enlargement in patients with MD and measured EH volume semi-quantitatively^11–13^. In this study, we longitudinally compared the change in EH morphology using Gd-enhanced inner ear MRI and inner ear function involving all semicircular canals (SCs) using caloric and video head impulse test between two groups. Regarding functional evaluation of MD, our previous longitudinal cohort study revealed that vertical semicircular canal deteriorated and hearing levels decreased during early stage of MD^12^. We discuss the effect of PPT regarding inner ear pathophysiology in MD and the significance of our findings with specific reference to known EH pathophysiology.

## Results

### Participant flow diagram

**Figure 1:**
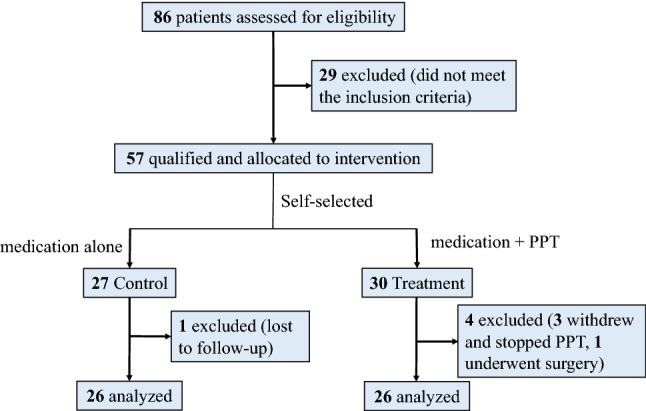
Participant flow diagram.

Participant enrollment is summarized in Fig. 1. The first participant was enrolled on July 20, 2020, and the last participant was enrolled on November 19, 2020. The final follow-up was on July 21, 2021. A total of 86 patients with repeated vertigo were screened for eligibility, and 57 of these met the inclusion criteria and were enrolled in the study. Twenty-seven participants received continuous medication only (Control group), while 30 participants self-selected to use the PPT device in addition to continuous medication (PPT group). One participant in the Control group was lost to follow-up. In the PPT group, three participants withdrew from the study, and one participant underwent surgery, resulting in 52 participants analyzed after 8 months. Before beginning PPT, no significant differences, except for durations and outcomes of caloric stimulus, were noted between the groups for the baseline demographics (Table 1).

**Table 1:** Baseline characteristics of the study participants by treatment group.

	Control group (n = 26)	PPT group (n = 26)	*P* value*
Age, mean (SD [range]), y	55.6 (15.6 [17–78])	54.7 (14.8 [22–84])	0.83 (*t*)
Female, %	19 (73.1)	13 (50)	0.09 (*χ*^2^)
Duration, median (25–75), months	33.5 (8.5–78.8)	61.5 (21.3–165)	0.04 (*U*)^a^
Vertigo, median (25–75), α/month	4 (2.75–10)	8 (1–14)	0.26 (*U*)
PTA thresholds, mean (SD [range]), dB	48.2 (24.3 [2.5–97.5])	48.0 (21.1 [5–88.8])	0.97 (*t*)
max-SPEV, mean (SD [range]), deg/s	21.1 (10.9 [3.5–41.5])	14.8 (9.3 [1–42.5])	0.03 (*t*)^a^
Diuretic use, %	23 (88.5)	20 (76.9)	0.27 (*χ*^2^)

*PPT* positive pressure therapy, *SD* standard deviation, *PTA* pure-tone audiometry, *max-SPEV* maximum slow phase velocity.

*Two-independent samples *t* test, *χ*^2^ test, or Mann–Whitney *U* test after evaluating normality and equal variances.

^a^Statistically significant difference.

### Frequency of vertiginous episodes and endolymphatic hydrops volume between the two groups

**Figure 2:**
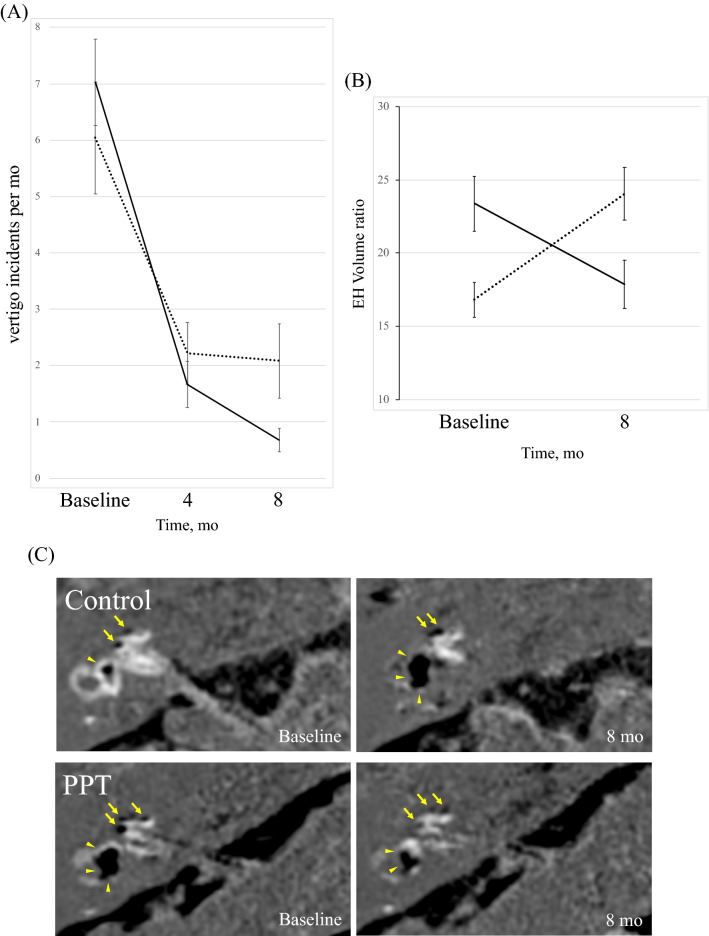
Changes in frequency of vertiginous episodes and Endolymphatic hydrops volume between two controlled groups. EH indicates endolymphatic hydrops, PPT indicates positive pressure therapy and MRI, magnetic resonance imaging. (**A**) Changes in incidents of vertigo between Control and PPT groups. (**B**) Change in volume ratio of total EH between Control and PPT groups. Error bars represent SE of the mean. Dotted line and solid line show Control group and PPT group, respectively. (**C**) Representative MRI. The black areas within the labyrinth represent EH. Yellow arrows and arrowheads indicate cochlear EH and vestibular EH, respectively. Upper half of image indicates mild EH in right cochlea and vestibule at baseline (upper half, left) and significant EH in right cochlea and vestibule 8 months later (upper half, right) in Control group. Lower half of image indicates significant EH in right cochlea and vestibular at baseline (lower half, left) and mild EH in right cochlea and vestibule 8 months later (lower half, right) in PPT group.

Figure 2A provides the total number of definitive vertigo attacks per month by group for the baseline month and the follow-up months. ANOVA-RM showed a significant difference between time points (*F*(2, 100) = 47.05, *P* < 0.001; partial *η*^2^ = 0.485). There were no differences between the groups (*F*(1, 50) = 0.272, *P* = 0.604; partial *η*^2^ = 0.005) and the interaction effects (*F*(2, 100) = 2.162, *P* = 0.136; partial *η*^*2*^ = 0.041). Multiple comparisons showed that the total number of definitive vertigo attacks per month decreased at 4 months (*P* < 0.001) and 8 months (*P* < 0.001) compared with baseline.

Figure 2B shows the change in EH volume by group for the baseline month and the follow-up months. ANOVA-RM showed a significant difference in the interaction effects (*F*(1, 50) = 42.145, *P* < 0.001; partial *η*^2^ = 0.457). There were no main effects by group (*F*(1, 50) = 0.009, *P* = 0.924; partial *η*^2^ = 0.000) and time (*F*(1, 50) = 0.775, *P* = 0.383; partial *η*^2^ = 0.015). Tests of simple main effects revealed that EH volume in the PPT group was significantly larger vs the Control group at baseline (*P* = 0.005) and significantly smaller vs the Control group at 8 months (*P* = 0.014). The EH volume change in representative patients with MD over the 8-month period are shown in Fig. 2C. The MRI results of the patient in the Control group indicated mild EH in the right cochlea and vestibule at baseline (upper row, left) and significant EH in the right cochlea and vestibule 8 months later (upper row, right). The MRI results of the patient in the PPT group indicated significant EH in the right cochlea and vestibule at baseline (lower row, left) and mild EH in the right cochlea and vestibule 8 months later (lower row, right).

### Auditory and vestibular function between the two groups

**Figure 3:**
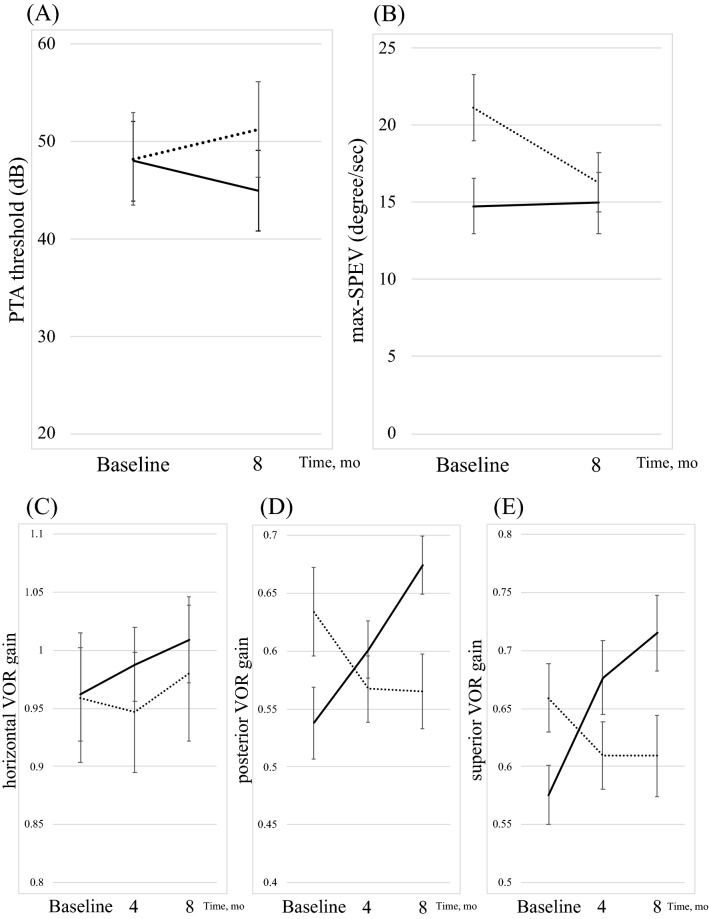
Serial changes in Auditory and Vestibular Function between two controlled groups. Error bars represent SE of the mean. Dotted line and solid line show Control group and PPT group, respectively. PTA indicates pure-tone audiometry, EH indicates endolymphatic hydrops and VOR, vestibulo-ocular reflex. (**A**) Change in PTA thresholds by group for the baseline month and the follow-up month. (**B**) Change in EH volume by group for the baseline month and the follow-up months. (**C**) Serial changes in horizontal VOR gain by group for the baseline month and the follow-up months. (**D**) Serial changes in posterior VOR gain by group for the baseline month and the follow-up months. (**E**) Serial changes in superior VOR gain by group for the baseline month and the follow-up months.

Figure 3A shows the change in PTA thresholds by group for the baseline month and the follow-up month. ANOVA-RM for PTA thresholds showed a significant difference in the interaction effects (*F*(1, 50) = 8.702, *P* = 0.005; partial *η*^2^ = 0.148). There were no main effects related to the group (*F*(1, 50) = 0.268, *P* = 0.607; partial *η*^2^ = 0.005) or time (*F*(1, 50) = 0.001, *P* = 0.981; partial *η*^*2*^ = 0.000). Tests of simple main effects revealed that PTA thresholds were not significantly different between the groups at 8 months (*P* = 0.333).

Figure 3b shows the change in max-SPEV by group for the baseline month and the follow-up month. ANOVA-RM for max-SPEV showed a significant difference in the interaction effects (*F*(1, 50) = 8.034, *P* = 0.007; partial *η*^2^ = 0.138) and between time points (*F*(1, 50) = 6.742, *P* = 0.012; partial *η*^2^ = 0.119), with no significant differences between the groups (*F*(1, 50) = 2.12, *P* = 0.152; partial *η*^2^ = 0.041). Tests of simple main effects revealed that the significant difference in max-SPEV between the groups (*P* = 0.028) decreased to non-significance at 8 months (*P* = 0.634).

Figure 3c shows the change in horizontal VOR gain by group for the baseline month and the follow-up months. ANOVA-RM for horizontal VOR gain showed no significant differences between time points (*F*(2, 100) = 0.969, *P* = 0.383; partial *η*^2^ = 0.019), between the groups (*F*(1, 50) = 0.194, *P* = 0.662; partial *η*^2^ = 0.004), and in the interaction effects (*F*(2, 100) = 0.218, *P* = 0.805; partial *η*^2^ = 0.004).

Figure 3d shows the change in posterior VOR gain by group for the baseline month and the follow-up months. ANOVA-RM for posterior VOR gain showed a significant difference in the interaction effects (*F*(2, 100) = 8.54, *P* < 0.001; partial *η*^2^ = 0.146). There were no main effects related to the group (*F*(1, 50) = 0.241, *P* = 0.626; partial *η*^2^ = 0.005) and time (*F*(2, 100) = 1.284, *P* = 0.281; partial *η*^2^ = 0.025). Tests of simple main effects revealed that posterior VOR gain in the PPT group increased significantly (*P* = 0.001). Multiple comparisons showed that the value of posterior VOR gain in the PPT group was larger vs the Control group at 8 months (*P* = 0.01).

Figure 3e shows the change in superior VOR gain by group for the baseline month and the follow-up months. ANOVA-RM for superior VOR gain showed a significant difference in the interaction effects (*F*(2, 100) = 16.477, *P* < 0.001; partial *η*^2^ = 0.248) and between time points (*F*(2, 100) = 3.367, *P* = 0.038; partial *η*^2^ = 0.063), with no significant differences between the groups (*F*(1, 50) = 0.598, *P* = 0.443; partial *η*^2^ = 0.012). Tests of simple main effects revealed that superior VOR gain in the PPT group increased significantly (*P* < 0.001). Multiple comparisons showed that the value of superior VOR gain in the PPT group was smaller than that in the Control group at baseline (*P* = 0.037) and larger vs the Control group at 8 months (*P* = 0.032).

Table 2 shows the time course changes in mean values for the outcome measures of the constructs over the 8-month period by group, and summarizes the calculated *p*-value obtained with ANOVA-RM.

**Table 2:** Changes in and comparison of outcome constructs between the control and PPT groups.

	Mean value (95% CI)						*P*-value of ANOVA-RM			ES
	Control group (n = 26)			PPT group (n = 26)			Group	Time	Group × time interaction	*η*^2^
	Baseline	4 months	8 months	Baseline	4 months	8 months				
Vα (/month)	6.0 (4.3–7.8)	2.2 (1.2–3.2)	2.1 (1.1–3.1)	7.0 (5.2–8.8)	1.7 (0.7–2.6)	0.7 (− 0.3–1.7)	0.604	< 0.01^a^	0.120	0.041
EH volume (%)	16.8 (13.7–19.9)		24.0 (20.6–27.5)	23.4 (20.2–26.5)		17.9 (14.4–21.3)	0.924	0.383	<0 .01^a^	0.457
PTA thresholds (dB)	48.2 (39.3–57.2)		51.3 (42.2–60.3)	48.0 (39.0–56.9)		45.0 (35.9–54.1)	0.607	0.981	0.005^a^	0.148
max-SPEV (deg/sec)	21.1 (17.1–25.1)		16.3 (12.4–20.2)	14.8 (10.8–18.7)		15,0 (11.1–18.9)	0.152	0.012^a^	0.007^a^	0.138
VORh	0.96 (0.86–1.1)	0.95 (0.86–1.0)	0.98 (0.88–1.1)	0.97 (0.87–1.1)	0.99 (0.90–1.1)	1.0 (0.91–1.1)	0.662	0.383	0.805	0.004
VORp	0.63 (0.56–0.70)	0.57 (0.51–0.62)	0.57 (0.51–0.62)	0.54 (0.47–0.61)	0.60 (0.55–0.66)	0.67 (0.62–0.73)	0.626	0.281	< 0.01^a^	0.146
VORs	0.66 (0.60–0.71)	0.61 (0.55–0.67)	0.61 (0.54–0.68)	0.58 (0.52–0.63)	0.68 (0.62–0.74)	0.72 (0.65–0.78)	0.443	0.038^a^	< 0.01^a^	0.248

*ANOVA-RM* two-factor repeated measures analysis of variance, *CI* confidence interval, *ES*, effect size, *Vα* number of attacks of vertigo per month, *EH* endolymphatic hydrops, *PTA* pure-tone audiometry, *max-SPEV* maximum slow phase velocity, *VORh* the value of horizontal vestibulo-ocular reflex (VOR) gain, *VORp* the value of posterior VOR gain, *VORs* the value of superior VOR gain.

^a^Statistically significant difference.

## Discussion

To our knowledge, this study represents the first clinical trial to clarify morphological changes of EH in vivo during PPT for patients with MD. The study met its primary outcome, which was to confirm that EH volume decreased with a reduction in vertigo attacks during PPT. The finding that 8 months of PPT has a limited effect on the control of vertigo is consistent with a recent meta-analysis^7^. The trend in vertigo reduction after the fourth month of PPT needs to be investigated further. Meanwhile, the change in EH volume was opposite between the two groups; EH volume in the Control group increased and that of the PPT group decreased. The finding that EH volume in active MD increased during medical therapy is consistent with previous studies^12,14^. The detailed mechanism underlying the effect of PPT is still unknown regarding whether PPT inhibits endolymph production or encourages endolymph drainage or both^15,16^. One possible explanation for the effect of PPT is that the pressure pulses promotes longitudinal endolymph flow^17^, which would result in a removal of saccular otoconial clumps and a reperfusion of endolymphatic obstruction^18^. Regardless, EH volume reduction during PPT is an hypothesized pathway for relieving MD symptoms, and this pathway was first demonstrated in a recent case report when combined with endolymphatic sac surgery^13^. Our study indicated that a therapeutic process occurring in the inner ear may be quite different between PPT treated and untreated groups although both groups achieved equivalent control of vertigo attacks.

Regarding cochlear function, the finding of no significant difference in hearing levels between the groups after PPT is consistent with a recent randomized clinical trial^19^. EH volume is reportedly correlated with hearing level in the affected ear^14^, and its shrinkage is associated with vertigo remission and hearing improvement^20^. The fact that decreased EH volume did not yield hearing improvement in this study might imply that irreversible degeneration of hearing had developed in the PPT group, which had a longer disease duration.

To our knowledge, few reports have examined the change in vestibular function before and after PPT. Regarding function in the horizontal SC, the PPT group, unlike the Control group, showed no significant functional deterioration. As caloric response deteriorates during the chronic course of MD^21^, PPT might have inhibited the course. Regarding function in the vertical SC, function deteriorated in the Control group, as reported previously^12^, and improved significantly in the PPT group. The finding that function in the vertical SC improved with decreased EH volume might mean that expansion of the endolymph duct causes decreased mobility of the cupula and subsequent malfunction in the vertical SC.

Our study has several limitations. This study was a non-randomized trial with self-selection of treatment, and the characteristics in the two groups were not fully matched. Patients with longer disease duration tended to self-select PPT even though this therapy required additional monthly costs; therefore, the results should be interpreted with caution. Another limitation of this study is that we did not use vestibular-evoked myogenic potentials to assess the function of the saccule and utricle. This would be necessary to fully characterize inner ear function in MD.

In conclusion, we confirm that EH volume decreased with a reduction in vertigo attacks when combined with PPT, while EH volume increased with medications alone. Regarding vertigo control, there was no difference between the groups; however, PPT achieved functional improvement in the vertical SC. The pathophysiological changes in the inner ear by self-administered PPT are quite different from those caused by medications alone.

## Methods

### Study design

This prospective, nonrandomized, controlled, single-center, clinical trial had two arms: a control group, which were prescribed an osmotic diuretic and betahistine mesilate at daily doses of 36 mg, and a treatment group, which self-selected to use a PPT device in addition to the same medications. The 8-month study period was established a priori as a reasonable time in accordance with the results of a previous study^13^. All participants provided written informed consent and received no stipend. The study was conducted in accordance with the Declaration of Helsinki. The trial protocol was approved by the Ethics Committee of Kansai Rosai Hospital (certificate number: 15B034e).

### Inclusion and exclusion criteria

Participants were required to be diagnosed as unilateral definitive MD according to the criteria of the AAO-HNS^22^, with repeat definitive vertigo attacks lasting more than 20 min of more than one per month for the 2 months prior to entering the study despite diet and lifestyle modifications. An additional entry criterion was a functionality level of 3 to 4^22^. We determined in advance whether the patients met the diagnostic criteria for vestibular migraine (VM), and if so, they were excluded. Participants were also excluded from the study if they had vestibular schwannoma, eardrum perforation or ear canal injury in the affected ear, or if they had bronchial asthma or renal impairment to cause side effects of contrast medium, or if they had metal implants in the body.

### Procedures

All participants underwent neuro-otological testing and Gd-enhanced inner ear MRI within 2 weeks following their first consultation at our institution. Participants were instructed to record the date, time, severity, and duration of vertigo attacks in a self-reporting diary^23^. We asked all participants to decide by the next consultation date in 2 weeks whether to receive PPT in addition to medications. PPT incurred an additional $50 monthly cost, determined by Japan’s public medical insurance system. The allocation to the additional PPT intervention was thus made by self-determination, and participants were then assigned to either the Control group or the PPT group, prescribed an osmotic diuretic isosorbide at daily doses of 63 mg, and then followed. Follow-up assessments were scheduled at monthly intervals. We performed audiometry once a month, video head impulse testing (vHIT) was performed every 4 months, and caloric testing and MRI were performed every 8 months. These tests were performed on the same day if tests coincided.

### Power calculation

Because EH volume was considered the most important outcome in this study, power calculations were completed for the primary outcome of change in EH volume. Setting the effect size of EH% change at 0.25, a total sample size of 46 was necessary to achieve 90% power to detect a clinically relevant effect. To allow for a dropout rate of 10%, the final sample size was 52 participants.

### Clinical tests

#### Self-reporting diary

Participants entered the maximum level of vertigo severity and PPT use in the diary. Vertigo-free days were scored as 0, and days with a mild attack were scored as 1. Moderately severe attacks lasting more than 20 min were scored as 2; severe attacks lasting an hour or more or accompanied by nausea or vomiting were scored as 3. A level 4 attack was the worst attack experienced to date. A definitive vertigo day was any day with a vertigo score of 2, 3, or 4. Use of the diary began with recording baseline symptom levels during the 2-month assessment period prior to entering the study.

#### MRI

MRI was performed as previously described^11^. Briefly, a standard dose (0.2 ml/kg) of gadoteridol (ProHance; Eisai Co., Ltd., Tokyo, Japan) was injected intravenously, and 4 h later, MRI was performed using a 3 Tesla MR imaging unit (Magnetom Verio; Siemens, Erlangen, Germany) equipped with a receive-only 32-channel phased-array coil. All participants underwent heavily T2-weighted MR cisternography (MRC) for the anatomical reference of total lymph fluid volume, and heavily T2-weighted three-dimensional fluid-attenuated inversion recovery with inversion times of 2250 and 2050 ms. HYDROPS imaging was used to depict EH; the sequence parameters were described previously^10^.

#### EH image evaluation

EH images were evaluated as previously described^11^. Briefly, the HYDROPS-Mi2 image was created by multiplying the MRC and HYDROPS images using a Digital Imaging and Communications in Medicine Viewing program (OsiriX, ver.9.5; Pixmeo SARL, Geneva, Switzerland)^24^. The regions of interest (ROIs) for the contouring of the cochlea and vestibule on the MRC were copied to the HYDROPS-Mi2 image. Using the OsiriX histogram function, we calculated the total number of pixels in the ROI and the number of pixels with negative signal intensity values, which represent areas of vestibular and cochlear EH, in the ROI^11^. The EH volume ratio (EH%) was computed as the ratio of the number of negative signal intensity pixels in the ROI divided by the total number of pixels in the ROI.

#### vHIT

Three-dimensional vHIT using a video oculography device (ICS Impulse; GN Otometrics, Taastrup, Denmark) was performed as previously described^11^. Individual vestibulo-ocular reflex (VOR) gains in each SC plane were automatically calculated using the device’s software (OTOsuite Vestibular software, v4.00 Build 1286; GN Otometrics).

### Caloric test

Bithermal caloric testing was performed as previously described^11,25^. Induced nystagmus was recorded using an electronystagmogram (NY-50 and NY-50S; RION, Tokyo, Japan), and the maximum slow phase velocity (max-SPEV) of the nystagmus was measured following each irrigation to determine the absolute value describing the function of the horizontal SC. The value of max-SPEV was calculated as the average of two values.

### Auditory evaluation

Hearing function was assessed using pure-tone audiometry (PTA) (Audiometer AA-78; RION, Tokyo, Japan) and was evaluated according to the four-tone average formulated by (a + b + c + d)/4, with a, b, c, and d indicating hearing levels at 0.5, 1, 2, and 3 kHz, respectively, according to the modified 1995 American Academy of Otolaryngology-Head and Neck Surgery Foundation criteria^22^.

### Positive pressure therapy

The PPT device (EFET01; Daiichi Medical Co., Ltd., Japan) delivers intermittent, complex pressure pulses to the middle ear, without ventilation tube insertion, within the range of − 0.65 to 1.2 kPa^26^. The modulation frequency is 7 Hz, and the pulse duration is 0.142 s. The PPT device works for 3 min and stops automatically, once started. Allocated participants self-administered this device at home three times daily (Supplementary Information).

### Statistical analysis

The primary outcome measure was the change in EH volume and the frequency of vertigo attacks per month. The predefined secondary outcome measures were hearing function (PTA thresholds) and vestibular function (max-SPEV and VOR gain). Demographic variables were compared between the groups using the χ^2^ test for dichotomous variables and two-tailed unpaired *t* tests or the nonparametric Mann–Whitney test, if a variable failed a normality test, for continuous variables. Two-factor repeated measures analysis of variance (ANOVA-RM) was used to determine the group difference for the outcome construct. Two or three time points were treated as the within-subjects factor (effect over time), and differences between the Control and PPT groups were treated as the between-subjects factor. When ANOVA-RM indicated that the group × time interaction was significant, tests of simple main effects were performed to determine which group differed significantly across the intervention period. The alpha level of the post-hoc analyses was adjusted using the Bonferroni method. All statistical significance tests were two-sided, and an alpha-level of 0.05 was considered statistically significant. Data were analyzed using IBM SPSS Statistics software, version 26.0 (IBM Corp.), and SAS JMP Discovery software, version 14.3.0 (SAS Institute). All data analysis was performed from March 22, 2021 to August 20, 2021.

## Supplementary Information

Supplementary Information.

## Data Availability

The datasets during and/or analyzed during the current study available from the corresponding author on reasonable request.
