# Supplementary material for: Assessment of inner ear morphology and function in response to local positive pressure for Ménière’s disease: a nonrandomized controlled trial
Source: Sci Rep. 2022 Dec 1;12:20782. doi: 10.1038/s41598-022-25321-z (PMC9715546; doi:10.1038/s41598-022-25321-z)
Supplement: Supplementary file 1 — Supplementary Information. [file 41598_2022_25321_MOESM1_ESM.pdf]

# Prospective, Single-Centre, Clinical trial to Evaluate Positive Pressure Therapy for Ménière’s disease

Chief Investigator: Munehisa Fukushima (Director, Department of Otolaryngology, Head and Neck Surgery, Kansai Rosai Hospital)

Version 1.1 Date: 07.23.2021

Contents

1. **Summary** ........ 1
2. **Background and Rationale** ........ 1
3. **Assessment and management of risk** ........ 1
3−1 Potential risk ........ 1
3−2 Risk Management ........ 1
4. **Objectives** ........ 1
4−1 Primary Objectives ........ 1
4−2 Secondary Objectives ........ 1
5. **Trial Design** ........ 1
6. **Study Settings** ........ 2
6−1 Site Selection ........ 2
6−2 Study Setting ........ 2
6−3 Study Duration ........ 2
7. **Eligibility Criteria** ........ 2
7−1 Study population ........ 2
7−2 Participant Inclusion Criteria ........ 2
7−3 Participant Exclusion Criteria ........ 2
7−4 Participant Stopping Criteria ........ 2
8. **Trial Procedures** ........ 3
8−1 Recruiment ........ 3
8−2 Informed Consent Process ........ 3
9. **Assessments and Follow up** ........ 3
10. **Treatment for adverse events** ........ 3
10−1 Responding to participants when an adverse event occurs ........ 3
10−2 Reporting of Serious Adverse Events ........ 4
11. **Data Management and Quality Assurance** ........ 3
12. **Cost Burden on Participants** ........ 3
13. **Statistical Considerations** ........ 4
13−1 Primary Outcomes ........ 4
13−2 Secondary Outcomes ........ 4
13−3 Statistical Analysis Plan (SAP) ........ 4
14. **Record Keeping and Archiving** ........ 4
15. **Changes to the Implementation Plan etc.** ........ 5
16. **Complying with the Declaration of Helsinki** ........ 5
17. **Monitoring Requirement for the Trial** ........ 5
18. **Research Funds and Conflicts of Interest** ........ 5
19. **Compensation for health problems and obtaining insurance** ........ 5
19−1 Compensation for health problems ........ 5
19−2 Obtaining liability insurance ........ 5
20. **Publication Policy** ........ 5
21. **References** ........ 5

## 1. Summary

The purpose of this study is to longitudinally assess the morphological and functional changes of inner ear in patients with active Ménière's disease (MD) between medications-alone group and positive pressure therapy (PPT) combined group by using inner ear gadolinium (Gd)-enhanced MRI and neuro-otological testings.

## 2. Backgroud and Rationale

Ménière's disease (MD) is clinically characterized by recurrent vertigo, fluctuating hearing loss and persistent tinnitus with a high lifetime prevalence especially in middle-aged. Medical managements such as lifestyle modifications and medications are the first-line therapy, but twenty percent of MD patients are refractory to medical therapy and suffer frequent vertigo attacks with progressive profound hearing loss.

For intractable MD patients, available options other than function-ablative procedures are pressure pulse treatment (PPT) or endolymphatic sac surgery.[1] MD is pathologically defined as the idiopathic endolymphatic hydrops (EH) in the inner ear and reducing EH is an hypothesized pathway in these two therapies; however, this has not been directly demonstrated for PPT. PPT using portable device is now a second-line therapy for intractable MD and a newly developped device[2] was approved by Japan's public medical insurance system in September 2018.

EH, an objective index of treatment effect, is currently easily visualized using 3-Tesla magnetic resonance imaging (MRI) after intravenous administration of gadolinium (Gd). Using this imaging method, we characterized the morphology of *in vivo* EH in MD patients and measured EH volume semi-quantitatively.[3,4] However, the hypothetical morphological changes of *in vivo* EH during PPT, i.e. the reduction of EH, has not yet to be well investigated.

## 3. Assessment and management of risk

3−1 Potential risk

1. Allergic reactions to MRI contrast agent
2. Transient nausea, vomiting, and worsening of vertigo at caloric testing.

3−2 Risk Management

Participants with asthma and kidney disease will be excluded from the study. Participants may be withdrawn from the study if they have prior reaction to contrast media.

## 4. Objectives

4−1 Primary Objectives

To assess the change of EH volume.

4−2 Secondary Objectives

To assess the frequency of vertigo attacks per months using self-reporting diary.

To assess hearing function by PTA thresholds once a month.

To assess vestibular function by max-SPEV and VOR gain every 4 months.

## 5. Trial Design

This is a single-centre, prospective, nonrandomized-controlled, clinical trial with two arms: a control group, which were prescribed an osmotic diuretic and betahistine mesilate, and a treatment group, which self-selected to use a PPT device in addition to the same medications.

## 6. Study Settings

6−1 Site Selection

This study will be conducted at Kansai Rosai Hospital (KRH).

6−2 Study Setting

All participants assessments will be performed at KRH.

6-3 Study Duration

Each participant will prescribe an osmotic diuretic at daily doses of 63 mg and receive audiometry once a month, video head impulse testing (vHIT) every 4 months, and caloric testing and MRI every 8 months for the duration of 36 months and will be followed up.

## 7. Eligibility Criteria

7-1 Study population

Participants were required to be diagnosed as unilateral definitive MD according to the criteria of the AAO-HNS,[5] with repeat definitive vertigo attacks of more than one per month for the 2 months prior to entering the study despite diet and lifestyle modifications. An additional entry criterion was a functionality level of 3 to 4. Fifty participants will be enrolled into the study.

7-2 Participant Inclusion Criteria

1) Male or female

2) No age limits

2) Confirmed diagnosis of Ménière’s disease at any time; according to the 1995 criteria of AAO-HNS.

7-3 Participant Exclusion Criteria

Participants were also excluded from the study if a or more of the following criteria are met:

1. Participants had bronchial asthma or renal impairment.
2. Participants met the diagnostic criteria for vestibular migraine.
3. Participants had eardrum perforation or ear canal injury in the affected ear.
4. Pregnant or breastfeeding.
5. Any clinically significant or unstable medical or surgical condition that in the opinion of the PI or PI-delegated clinician may put the participant at risk when participating in the study or may influence the results of the study or affect the participant’s ability to take part in the study as determined by medical history, physical examinations, or laboratory tests.

7-4 Participant Stopping Criteria

1) Participants withdrawal of consent
2) Non-compliance with the protocol
3) Occurrence of change in medical status or an incidental finding that leads the investigator concerned about the participant’s welfare
4) Inability to continue, loss to follow-up
5) The termination of the study by the Chief/Principal Investigator (CI/PI).

If enrolled participants prematurely discontinue the study or do not comply with the protocol, additional participants may be enrolled as replacement participants and assigned to the same treatment sequence at the discretion of the Principal Investigator.

## 8. Trial Procedures

All participants underwent neuro-otological testing and Gd-enhanced inner ear MRI within 2 weeks following their first consultation at our institution. Participants were instructed to record the date, time, severity, and duration of vertigo attacks in a self-reporting diary.[6] We asked all participants to decide by the next consultation date in 2 weeks whether to receive PPT in addition to medications. The allocation to the additional PPT intervention was made by self-determination, and participants were then assigned to either the Control group or the PPT group, prescribed an osmotic diuretic at daily doses of 63 mg, and then followed.

8-1 Recruiment

Potential participants will be approached by their treating clinician (who in the case of this study may also be the CI/PI, Co-Investigator or PI-delegated clinician) during routine standard of care outpatient appointments at KRH. Potential participants will be given a participant information sheet (PIS) and the opportunity to ask questions before deciding whether or not to take part.

8-2 Informed Consent Process

It is the responsibility of the CI/PI Investigator, or a PI-delegated clinician to obtain written informed consent from each participant prior to participation in the trial, following adequate explanation of the aims, methods, anticipated benefits and potential hazards of the trial. “Adequate time” must be given for consideration by the participant before taking part. Consent will be sought at least 24 hours after being given the study documentation. It must be recorded in the medical notes when the PIS have been given to the participant. A copy of the signed informed consent form will be given to the participant. The original signed form will be retained in the trial file at site and a copy placed in the medical notes. Participants are under no obligation to enter the trial and can withdraw at any time during the trial without providing a reason.

The PIS and consent form will be reviewed and updated if necessary throughout the trial (e.g. where new safety information becomes available) and participants will be re-consented as appropriate.

## 9. Assessments and Follow up

Follow-up assessments were scheduled at monthly intervals. We performed audiometry once a month, video head impulse testing (vHIT) was performed every 4 months, and caloric testing and MRI were performed every 8 months. These tests were performed on the same day if tests coincided.

| Assessment | First consultation | 1m | 2m | 3m | 4m | 5m | 6m | 7m | 8m |
|---|---|---|---|---|---|---|---|---|---|
| Audiometry | ○ | ○ | ○ | ○ | ○ | ○ | ○ | ○ | ○ |
| vHIT | ○ | | | | ○ | | | | ○ |
| Caloric test | ○ | | | | | | | | ○ |
| MRI | ○ | | | | | | | | ○ |

## 10. Treatment for adverse events

10-1 Responding to participants when an adverse event occurs

When an adverse event takes place, the people conducting the research will take appropriate action immediately and, whenever necessary, quickly provide transport to a medical facility. A record of this will be taken without contradiction in a case report form. Whenever treatment for an adverse event becomes necessary, this will be conveyed to the participants.

10-2 Reporting of Serious Adverse Events

All serious adverse events that occur during the study period and all serious adverse events that occur after the termination (discontinuation) of the study and are suspected of having a connection to the study will be reported. When an adverse event is confirmed to have occurred, a report will immediately be provided to a supervisor and CI/PI.

## 11. Data Management and Quality Assurance

Utmost consideration will be given to protecting the participants' confidentiality when documenting raw data and handling consent forms pertaining to the study. The results of the study, when published, will not contain any information that could be used to identify the participants. It will be mentioned that the data on the participants obtained during the study shall not be used for any purpose other than the objectives of the study. The participants' privacy will be kept confidential and consideration will be given to ensure that absolutely no personal information be leaked. The research representative is responsible for managing the data. Anonymized data will be disclosed, but individual data will not be published.

A trial specific data management will be in place for the trial. This will contain details of the software to be used for the database, the process of database design, data entry, data quality checks, data queries, data security, database lock.

## 12. Cost Burden on Participants

The participants shall bear none of the costs required in this study and received no stipend. However, Tests or drugs required to treat the primary disease and complicating diseases shall not be borne by the present study.

## 13. Statistical Considerations

13-1 Primary Outcomes

The primary outcomes in this study are:

To assess the change of EH volume and the frequency of vertigo attacks per months.

13-2 Secondary Outcomes

The secondary outcomes in this study are:

Hearing function (PTA thresholds)

Vestibular function (max-SPEV and VOR gain).

13-3 Sample Size Calculation

The sample size has been selected by the chief investigator and is based on formal statistical calculation or consideration.

13-3 Statistical Analysis Plan (SAP)

Full details of all planned statistical analyses will be included in a written Statistical Analysis Plan which will be completed before the final study database is locked for analysis. The main focus of all analyses will be on the group difference for the outcome construct, and statistical significance tests will be used sparingly.

## 14. Record Keeping and Archiving

Essential documents relating to the conduct of the study are kept in the Department of Otolaryngology and Head and Neck Surgery, Kansai Rosai Hospital. The CI is responsible for their storage. The documents will be kept for a minimum of five years after the termination of the study period. Tables corresponding to the participants' identities will be kept separately and anonymized. Documents will be disposed of by shredder

following the end of the storage period.

## 15. Changes to the Implementation Plan etc.

In the event that there are any changes to the implementation plan or the informed consent form, the advanced approval of the ethical review committees will be required.

## 16. Complying with the Declaration of Helsinki

This study will be conducted in compliance with the Declaration of Helsinki.

## 17. Monitoring Requirement for the Trial

A trial specific oversight and monitoring plan will be established for this study.

## 18. Research Funds and Conflicts of Interest

The present study will be conducted using the medical research fund of the Hyogo Medical Association and by research funds to promote the hospital functions of Japan Organization of Occupational Health and Safety. The planning, implementation, and reporting of the present study will be free of any "potential conflicts of interest" that would affect the results of the study or the interpretation of the results, and the rights and interests of the participants will not be neglected in the implementation of the study.

## 19. Compensation for health problems and obtaining insurance

19-1 Compensation for health problems

No compensation will be offered for losses caused by any lawful act.

19-2 Obtaining liability insurance

To prepare for any liability, the research staff will be insured against liability.

## 20. Publication Policy

The anonymized data will be put through a statistical analysis and published sequentially. The analysis results will be disclosed in the form of papers, conference presentations, reports, and mass media publications.

## 21. References

1. Sajjadi H, Paparella MM. Meniere's disease. *Lancet.* 2008;372(9636):406-414.
2. Watanabe Y, Shojaku H, Junicho M, et al. Intermittent pressure therapy of intractable Meniere's disease and delayed endolymphatic hydrops using the transtympanic membrane massage device: a preliminary report. *Acta Otolaryngol.* 2011;131(11):1178-1186.
3. Fukushima M, Kitahara T, Oya R, et al. Longitudinal up-regulation of endolymphatic hydrops in patients with Meniere's disease during medical treatment. *Laryngoscope investigative otolaryngology.* 2017;2(6):344-350.
4. Fukushima M, Oya R, Nozaki K, et al. Vertical head impulse and caloric are complementary but react opposite to Meniere's disease hydrops. *Laryngoscope.* 2019;129(7):1660-1666.
5. Committee on Hearing and Equilibrium guidelines for the diagnosis and evaluation of therapy in Meniere's disease. American Academy of Otolaryngology-Head and Neck Foundation, Inc. *Otolaryngology--head and neck surgery : official journal of American Academy of Otolaryngology-Head and Neck Surgery.* 1995;113(3):181-185.

6. Fukushima M, Akahani S, Inohara H, Takeda N. Stability of Endolymphatic Hydrops in Meniere Disease Shown by 3-Tesla Magnetic Resonance Imaging During and After Vertigo Attacks. *JAMA Otolaryngol Head Neck Surg.* 2019;145(6):583-585.

Appendix 1

Self-check diary

# Self-check diary Date

______MM, YYYY

Name:________________________________ ID:_________________ Start date : DD________MM______YYYY_________

| 1 | 2 | 3 | 4 | 5 | 6 | 7 |
|---|---|---|---|---|---|---|
| Level of vertigo: | Level of vertigo: | Level of vertigo: | Level of vertigo: | Level of vertigo: | Level of vertigo: | Level of vertigo: |
| Stress level: | Stress level: | Stress level: | Stress level: | Stress level: | Stress level: | Stress level: |
| Stress level:: | Activity Level: | Activity Level: | Activity Level: | Activity Level: | Activity Level: | Activity Level: |
| 8 | 9 | 10 | 11 | 12 | 13 | 14 |
| Level of vertigo: | Level of vertigo: | Level of vertigo: | Level of vertigo: | Level of vertigo: | Level of vertigo: | Level of vertigo: |
| Stress level: | Stress level: | Stress level: | Stress level: | Stress level: | Stress level: | Stress level: |
| Activity Level: | Activity Level: | Activity Level: | Activity Level: | Activity Level: | Activity Level: | Activity Level: |
| 15 | 16 | 17 | 18 | 19 | 20 | 21 |
| Level of vertigo: | Level of vertigo: | Level of vertigo: | Level of vertigo: | Level of vertigo: | Level of vertigo: | Level of vertigo: |
| Stress level: | Stress level: | Stress level: | Stress level: | Stress level: | Stress level: | Stress level: |
| Activity Level: | Activity Level: | Activity Level: | Activity Level: | Activity Level: | Activity Level: | Activity Level: |
| 22 | 23 | 24 | 25 | 26 | 27 | 28 |
| Level of vertigo: | Level of vertigo: | Level of vertigo: | Level of vertigo: | Level of vertigo: | Level of vertigo: | Level of vertigo: |
| Stress level: | Stress level: | Stress level: | Stress level: | Stress level: | Stress level: | Stress level: |
| Activity Level: | Activity Level: | Activity Level: | Activity Level: | Activity Level: | Activity Level: | Activity Level: |
| 29 | 30 | 31 | | | | |
| Level of vertigo: | Level of vertigo: | Level of vertigo: | | | | |
| Stress level: | Stress level: | Stress level: | | | | |
| Activity Level: | Activity Level: | Activity Level: | | | | |

| Score | Level of vertigo : | Stress level: : | Stress level: : |
|---|---|---|---|
| | 0 : Vertigo-free | 0 : free | 0 : no reduction |
| | 1 : a mild attack | 1 : mild | 1 : minor reduction |
| | 2: Moderately severe attacks | 2 : moderately | 2 : moderate reduction |
| | 3 : severe attacks | 3 : severe | 3 : stay at home |
